# Data Fitting to Study Ablated Hard Dental Tissues by Nanosecond Laser Irradiation

**DOI:** 10.1371/journal.pone.0156093

**Published:** 2016-05-26

**Authors:** Y. Al-Hadeethi, S. Al-Jedani, M. A. N. Razvi, A. Saeed, A. M. Abdel-Daiem, M. Shahnawaze Ansari, Saeed S. Babkair, Numan A. Salah, A. Al-Mujtaba

**Affiliations:** 1 Physics Department, Faculty of Science, King Abdulaziz University, Jeddah 21589, Saudi Arabia; 2 Lithography in Devices Fabrication and Development Research Group, DSR, King Abdulaziz University, Jeddah 21589, Saudi Arabia; 3 Thamar University, Thamar, Yemen; 4 Physics Department, Faculty of Science, Zagazig University, Zagazig, Egypt; 5 Centre of Nanotechnology, King Abdulaziz University, Jeddah 21589, Saudi Arabia; University of Maribor, SLOVENIA

## Abstract

Laser ablation of dental hard tissues is one of the most important laser applications in dentistry. Many works have reported the interaction of laser radiations with tooth material to optimize laser parameters such as wavelength, energy density, etc. This work has focused on determining the relationship between energy density and ablation thresholds using pulsed, 5 nanosecond, neodymium-doped yttrium aluminum garnet; Nd:Y_3_Al_5_O_12_ (Nd:YAG) laser at 1064 nanometer. For enamel and dentin tissues, the ablations have been performed using laser-induced breakdown spectroscopy (LIBS) technique. The ablation thresholds and relationship between energy densities and peak areas of calcium lines, which appeared in LIBS, were determined using data fitting. Furthermore, the morphological changes were studied using Scanning Electron Microscope (SEM). Moreover, the chemical stability of the tooth material after ablation has been studied using Energy-Dispersive X-Ray Spectroscopy (EDX). The differences between carbon atomic % of non-irradiated and irradiated samples were tested using statistical t-test. Results revealed that the best fitting between energy densities and peak areas of calcium lines were exponential and linear for enamel and dentin, respectively. In addition, the ablation threshold of Nd:YAG lasers in enamel was higher than that of dentin. The morphology of the surrounded ablated region of enamel showed thermal damages. For enamel, the EDX quantitative analysis showed that the atomic % of carbon increased significantly when laser energy density increased.

## Introduction

In dentistry, cavity preparation is carried out through either a low-speed high-torque handpiece or a high-speed low-torque handpiece [1]. Despite their efficiency, they are still somewhat painful, irritating, and noisy [2]. In addition, it is necessary to use local anesthetic for the majority of dental procedures. A continuous water spray is used in conjunction with the drills to balance the temperature produced by friction between the drill and the tooth. In addition, dentin contains a large number of nerve endings that are connected with the pulp [3]. The dentin pain mainly arises from a rapid outward flow of fluid in dentinal tubules [4].

Lasers have been used in dentistry since 1964 [5] as complementary methods. They have been developed significantly for various dental applications [6,7]. Using laser in dentistry is comfortable almost painless as compared with conventional drilling systems. Therefore, the use of lasers in dentistry has increased markedly over the past few decades. They have been reported as potential tools for dentistry in many literatures [8–16]. Using lasers to ablate hard tissue may cause cracks and fractures, because they are prone to both shear and compressive stresses [17, 18]. Despite these risks, they are still an attractive alternative. The risks of lasers can be avoided using the optimum parameters of laser hard tissue interaction. Therefore, determining the laser parameters such as power density, wavelength, exposure time, repetition rate, and spot size [19, 20] is substantial. The evolution of using lasers in dental hard tissue ablation can be found in many studies [8, 10, 21–29]. These studies were concerned on using different lasers with different pulse durations in dental hard tissue ablation. However, only few studies were performed to estimate the nanosecond (ns), neodymium-doped yttrium aluminum garnet; Nd:Y_3_Al_5_O_12_ (Nd:YAG) laser ablation thresholds for both enamel and dentin tissues. Furthermore, 5 ns pulse durations ensure the minimum possible heating and consequently excluded damage to the dental hard tissues as compared to picosecond (ps) and femtosecond (fs) pulse durations. In addition, there is insufficient information regarding the ns, Nd:YAG laser ablation for both enamel and dentin. In this field, most of the studies focused on certain type of lasers such as Erbium family or Ti: Sapphire lasers.

Using data fitting, this study aimed to determine the enamel and dentin ablation thresholds of human teeth using pulsed, 5 ns, Nd:YAG laser at 1064 nm. In this work, laser-induced breakdown spectroscopy (LIBS) technique was utilized. LIBS is considered as an atomic emission spectroscopy, a highly energetic pulsed laser where the laser is used as the excitation source. The plasma formation occurs when laser beam with sufficient irradiance (Wcm^-2^) atomizes and excites samples. In addition, this study investigated the changes in the morphology and chemical structure of the dental hard tissues following laser irradiation using Scanning Electron Microscope (SEM) and Energy-Dispersive X-ray Spectroscopy (EDX).

## Materials and Methods

### Ethics statement

This study was approved by Research Ethic Committee of King Abdulaziz University Hospital and according to the ethical guidelines of the Declaration of Helsinki. Written informed consent was obtained from patients. Extracted Sound teeth were collected from them during the course of orthodontic treatment.

### Teeth samples

Fifty extracted sound human teeth, (non-carious third molars and premolars) for orthodontic purposes were used. Teeth were cleaned from adherent materials using water and ultrasonic unit [30]. For sterilization, samples were placed in sodium hypochlorite for 5 minutes, and then they were kept in phosphate-buffered saline (PBS) with 0.02% thymol to inhibit bacterial growth until samples were used [31]. The samples were prepared as slices and then polished in order to satisfy the experimental requirements according to reported protocols [9, 32]. A number of slices parallel to the occlusal surface were processed using a water-cooled low-speed diamond wheel saw model 650 (SBT INC., CA, USA). Every slice thickness was 2 mm. For polishing, surfaces of a sample were mechanically polished using 1500 grit silicon carbide a wet sandpaper [32] before laser irradiation to increase the measurement accuracy. Finally, samples were cleaned for 10 minutes [33] in an ultrasonic bath to remove conjoined particles.

Teeth slices were divided randomly into two main groups. The first main group (30 teeth) was irradiated by 3 shots with different laser energy densities (3 teeth/energy density). The second main group (20 teeth) was divided randomly into 4 subgroups (5 teeth/group). Three of the subgroups were irradiated by 100 shots with three different laser energy densities of 5.6, 11.2, and 17.2 J/cm^2^ (1 subgroup/energy density). The fourth subgroup was used as non- irradiated group. Then, all subgroups of second main group were studied by EDX.

### Laser system

In this study, the laser system that has been used, was ULTRA CFR Nd:YAG (Big Sky, USA). It operates at wavelength of 1064 nm, delivers Q-switched pulses at of approximately 5 ns durations, repetition rates of 1 to 20 Hz, a maximum average power output of 600 mW, and pulse stability of ±3%. System allowed independent adjustment of three variables: average power, repetition rate, and power density‏. The laser beam was focused onto the slice surfaces using a 7.5 cm focal length Plano convex lens. The laser energy density distribution over the surface at the irradiation spot is close to elliptical with diameters of 316 and 587 μm on the sample. The energy density varies from 1.2 to 26.39 J/cm^2^.

### LIBS method

LIBS were used to determine the ablation thresholds for both enamel and dentin. The Nd:YAG laser pulses were focused on teeth samples to induce plasmas. The LIBS system that was used is LIBS/3000 (Ocean Optics, USA). It consists of seven of the HR3000 high-resolution miniature fibre optic spectrometers. The spectrometer is of Czerny-Turner type. The spectrometers are triggered to acquire and read out data simultaneously. The response of HR3000 is in the range of 200–1100 nm with 0.1 nm spectral resolution. The spectra of samples were recorded at different energy densities. The best detector delay was optimized in the range of 2.5 μs. The emission intensities (100–900 nm) were collected using a fibre optic bundle connected to linear charge-coupled device (CCD) array spectrometers. The sample chamber is a plastic box equipped with safety inter-lock to stop laser system operation when chamber door is opened. It is also equipped with manually adjustable sample stage, which translates in two dimensions X and Y. The laser pulse entered the chamber from the top and was focused on the tooth slice with a 7.5 cm focal length lens, which was adjustable along the vertical axis. The emission collection optic was held in a fixed position relative to a focused lens and translates vertically with the lens.

### Morphological and elemental tests

The morphological changes were characterized using Field Emission Scanning Electron Microscope (FESEM) model Zeiss Ultra60 (Carl Zeiss, Jena, Germany). SEM was used to get clear observations after laser irradiation. In SEM, the sample must be electrically conductive; otherwise, the surface of the sample has to be coated with a very thin film using materials such as gold or platinum to reduce the charging in the sample. To avoid this problem, energy filter (r-filter) was used to make it possible to observe the surface morphology and the nano-structures’ morphology. Gentle beam (GB) mode was used for decelerating the incident electrons just before they hit the sample and for reducing the incident electron penetration and the charging in the sample. The GB mode gives high-resolution images without damaging the sample surface [34]. Moreover, EDX analysis was accomplished to detect the changes in the elemental composition after irradiating the teeth by laser.

### Data fitting and statistical analysis

Calcium lines that appeared in LIBS spectra were chosen as the indictors for occurring the ablation. They appeared with different wavelengths. The areas of these peaks were calculated and were plotted with respect to the laser energy densities. Then, data fitting was used to represent the relation between peak areas and energy densities. From the data fitting, the ablation thresholds were determined.

The carbon atomic % of non-irradiated and irradiated samples that were investigated by EDX technique were displayed with their mean value ± standard error of the mean (SE). In addition, the differences between carbon atomic % of non-irradiated and irradiated samples were tested using the t-test for unpaired measurements. P < 0.05 was considered as significant. All of the peak areas calculations, the data fitting, and statistical analysis were performed using ORIGIN 8.5 software (OriginLab Corporation, Massachusetts, USA).

## Results

The ablation threshold is generally defined as the minimum energy density that atom needs to escape from the material [35]. Therefore, ablation threshold was defined as appearance of minimal intensity of calcium line by using LIBS. Enamel and dentin tissues were ablated with Nd:YAG laser operating at 1064 nm, with a 5 ns pulse duration, and 1Hz repetition rates. When a high power pulsed-laser beam hits the target, it leads to local heating and evaporation‏ ‏of the sample materials. The ablated materials form the plasma plume, which expand in the ambient atmosphere [36]. From this point, there is a relationship between the plasma intensity and the ablated material. Using higher laser energy leads to obtain higher plasma intensity and ablated material. LIBS allows to define both the element in the sample and plasma intensity. The intensity of emission line is proportional to the number density of emitters, which is proportional to the concentration of the emitter in the irradiated‏ ‏sample [37]. Therefore, the emission intensity is linearly correlated to the concentration of given species‏ ‏in the sample [37].

**Fig 1:**
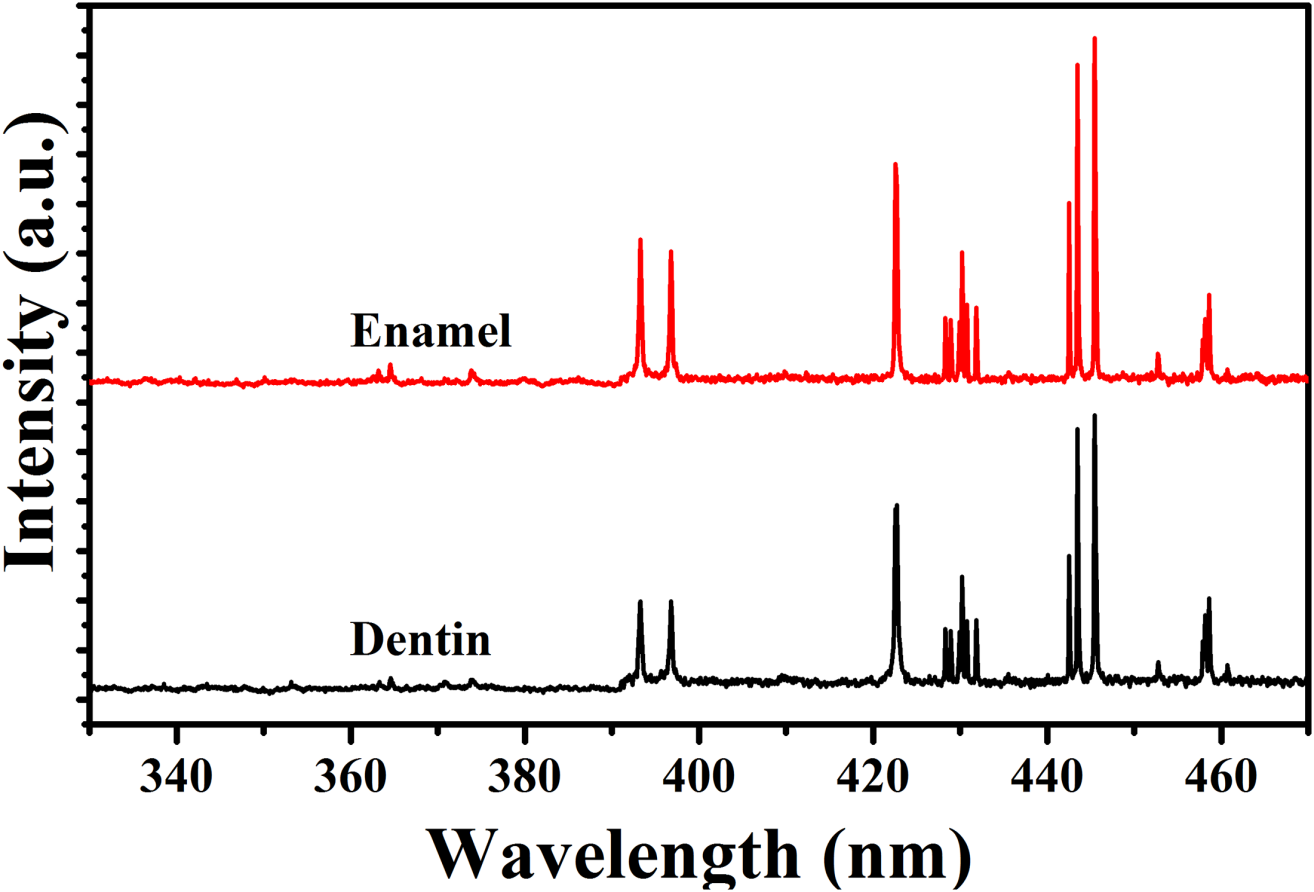
LIBS spectra of enamel and dentin laser ablation. The intensity in arbitrary units (a.u.) of calcium lines in the LIBS spectra of enamel and dentin Nd:YAG laser ablation.

**Fig 2:**
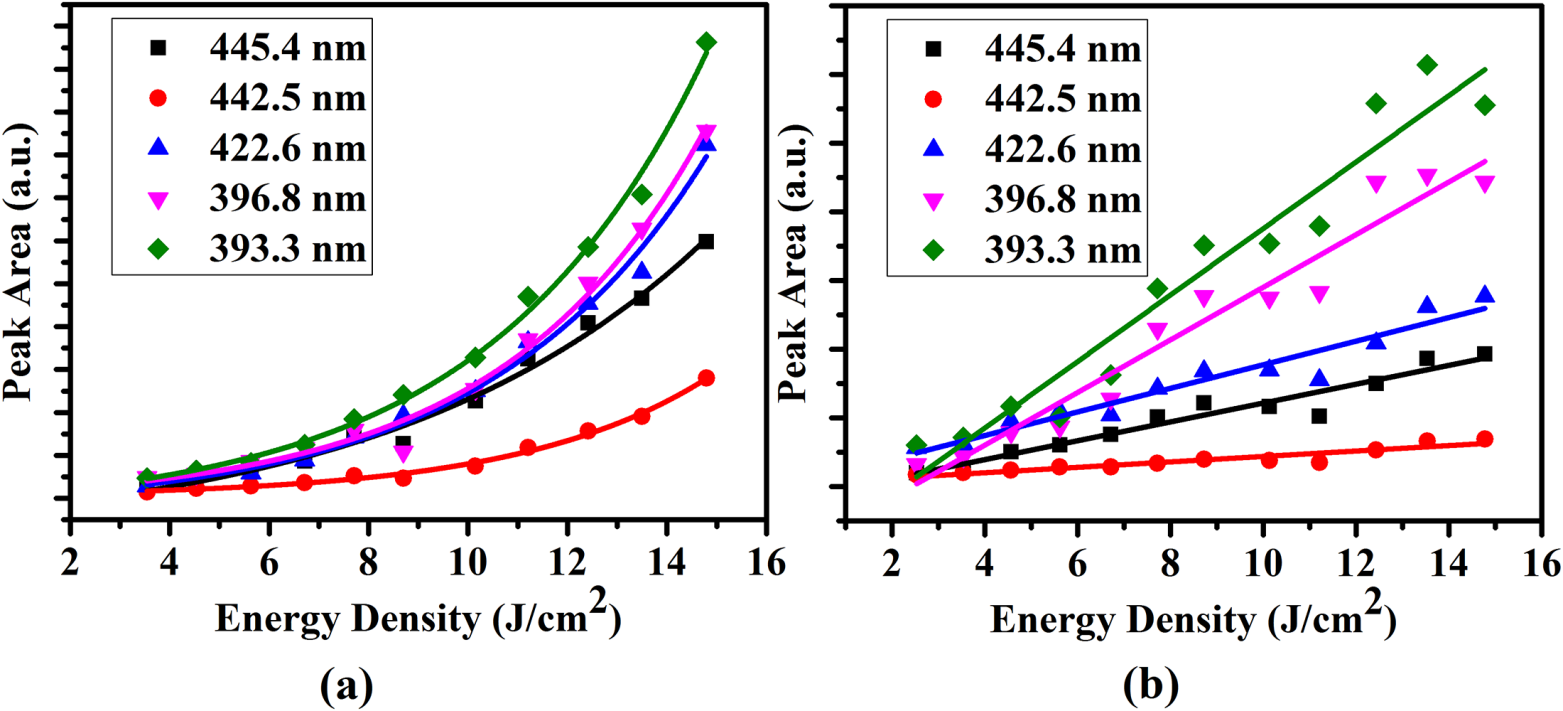
The relation between the peak areas of calcium lines and energy densities. (a) calcium line peak area versus energy density for enamel (b) calcium line peak area versus energy density for dentin.

Fig 1 shows the enamel and dentin LIBS spectra. These spectra reveal that calcium lines appeared at 393.3, 396.8, 422.6, 442.5, and 445.4 nm. The peak areas of these lines were calculated at different energy densities for both enamel and dentin tissues. The relation between the peak areas of calcium lines and energy densities for both enamel and dentin are represented in Fig 2(a) and 2(b), respectively.

**Fig 3:**
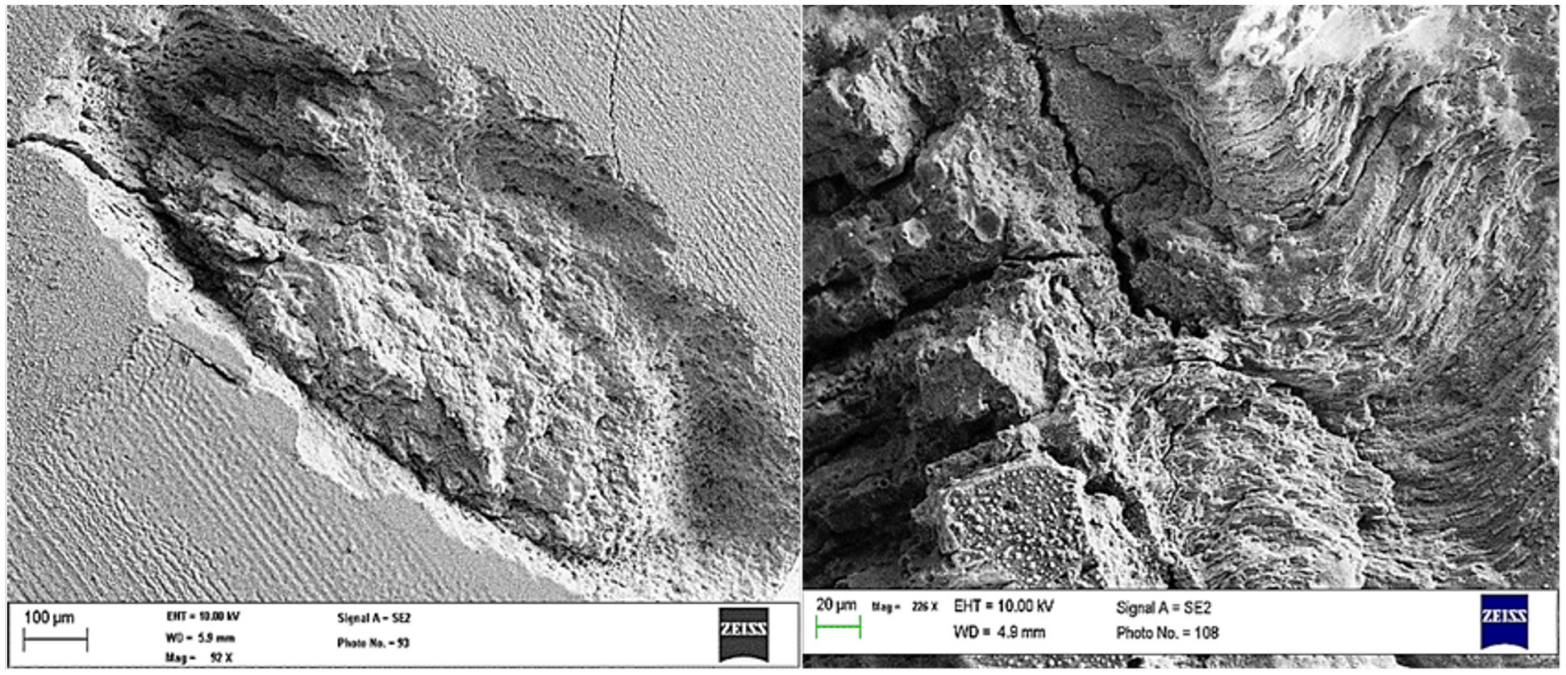
SEM images. Enamel surface after ablation with Nd:YAG laser at energy density of 17.2 J/cm^2^ and 100 shots.

**Fig 4:**
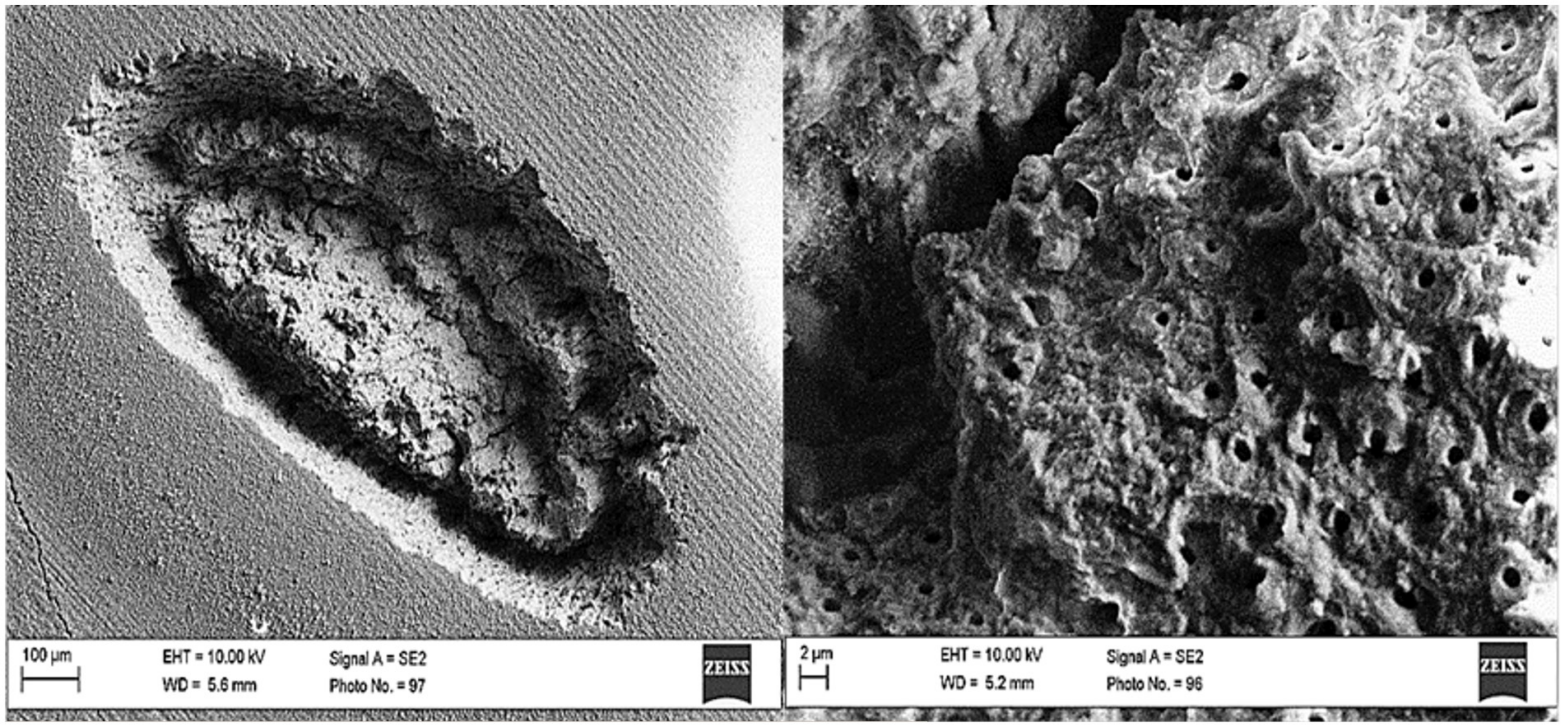
SEM images. dentin surface after ablation with Nd:YAG laser at energy density of 11.2 J/cm^2^ and 100 shots.

Fig 3 shows SEM images of enamel surface top view that was irradiated using laser energy density of 11.2 J/cm^2^ at 100 shots, while SEM images of the top view of dentin surface that was irradiated using laser energy density of 17.2 J/cm^2^ at100 shots are shown in Fig 4.

**Fig 5:**
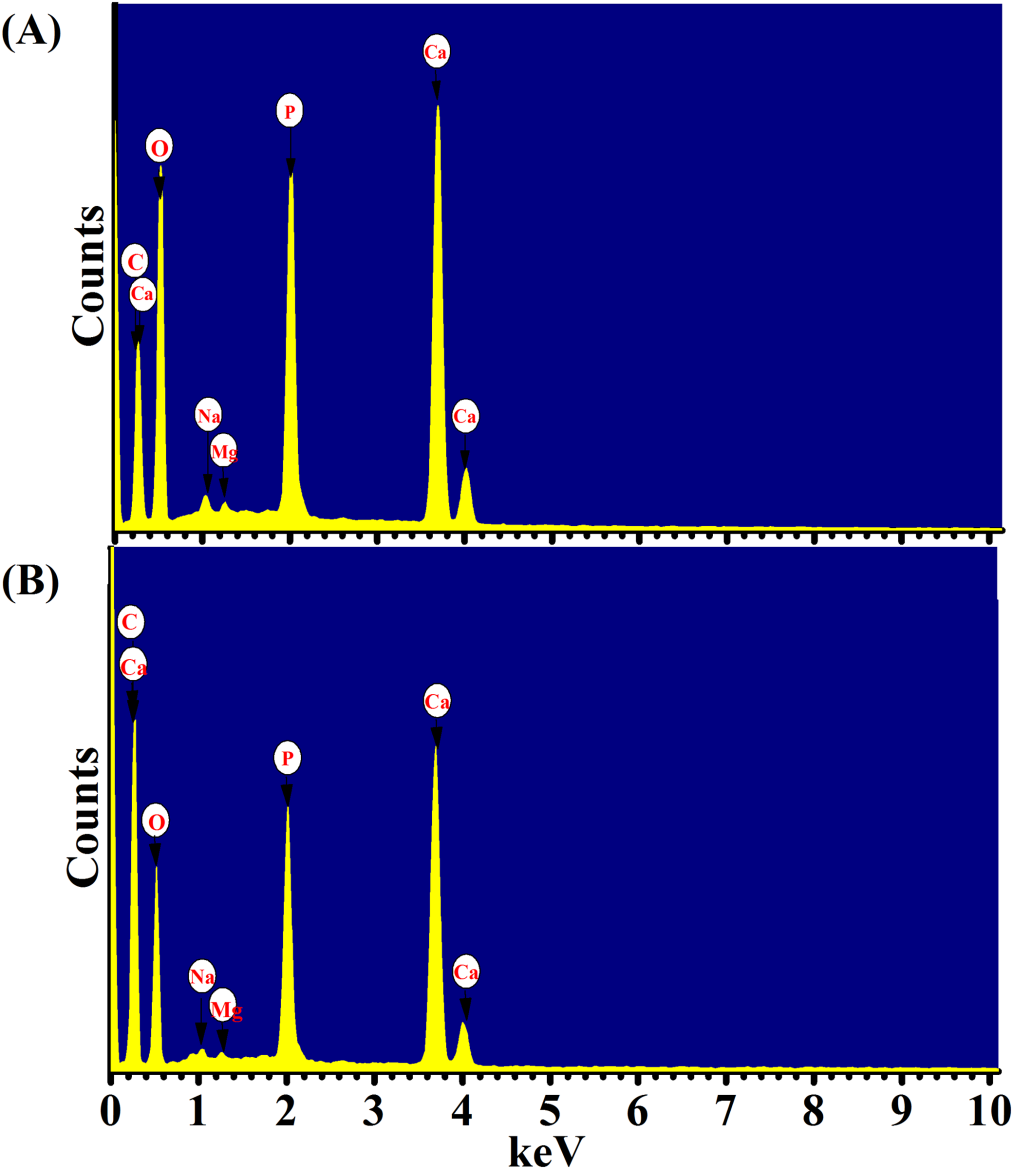
EDX spectra. (A) EDX spectrum of non-irradiated enamel tissue. (B) EDX spectrum of irradiated enamel tissue at energy density of 11.2 J/m^2^.

Elements atomic % of dentin and enamel were studied using EDX. Since, a hydroxyapatite compound contains elements with low atomic numbers, such as Ca, P, and O. Fig 5 displays the EDX spectra of non-irradiated enamel tissue and irradiated enamel tissue by energy density of 11.2. Six elements (Ca, P, O, C, Na, and Mg) can be noticed in the EDX spectra. In addition, Fig 5 shows increasing in the peak of carbon element in the irradiated samples compare to the non-irradiated samples. Table 1 lists the elements atomic % of non-irradiated and irradiated enamel tissues using energy densities of 5.6, 11.2, and 17.2 J/cm^2^, while Table 2 lists the elements atomic % of non-irradiated and irradiated dentin tissues using the same energy densities. Na and Mg were neglected because of their low concentrations. Thus, they were not listed in Tables 1 and 2. For enamel tissues with tested energy densities, Table 1 shows that there were significant decreases in the element atomic % of O, Ca, P, and C elements. In addition, there was a significant tandem increase in the atomic % of C in enamel tissues with increasing the energy density. On other hand, there were no significant changes in all of the elements atomic % with respect to dentin tissues when energy density of 5.6 J/cm^2^ was used for ablation.

**Table 1:** Elements atomic % for non-irradiated and irradiated enamel tissue at energy densities of 5.6, 11.2, and 17.2 J/cm^2^.

Elements	Non-irradiated	5.6 (J/cm^2^)	11.2 (J/cm^2^)	17.2 (J/cm^2^)
C	19.46 ± 1.53	34.05 ± 2.09^a^	47.19 ± 4.59^a^	55.88 ± 4.08^a^
O	56.10 ± 3.01	49.87 ± 1.95^a^	40.83 ± 6.05^a^	33.92 ± 2.67^a^
P	8.07 ± 0.56	5.04 ± 0.37^a^	3.02 ± 0.11^a^	2.45 ± 0.09^a^
Ca	16.37 ± 3.64	11.04 ± 0.91^a^	8.96 ± 0.39^a^	7.75 ± 1.52^a^

Parameters (mean ± SD).

^a^ significant change to the non-irradiated values (p < 0.05).

**Table 2:** Elements atomic % for non-irradiated and irradiated dentin tissue at energy densities of 5.6, 11.2, and 17.2 J/cm^2^.

Elements atomic %	Non-irradiated	5.6 (J/cm^2^)	11.2 (J/cm^2^)	17.2 (J/cm^2^)
C	19.89 ± 2.13	23.31 ± 4.22	27.56 ± 4.66^a^	29.85 ± 7.76^a^
O	61.91 ± 6.52	60.32 ± 4.17	58.04 ± 6.32	55.96 ± 3.42^a^
P	6.21 ± 0.53	5.58 ± 0.68	5.04 ± 0.87^a^	5.03 ± 0.86^a^
Ca	11.99 ± 2.91	10.79 ± 2.46	9.36 ± 3.19	9.16 ± 0.91^a^

Parameters (mean ± SD).

^a^ significant change to the non-irradiated values (p < 0.05).

The calculated percentages of calcium to phosphorus (Ca/P ratio) for non-irradiated and irradiated enamel and dentin tissues at energy densities of 5.6, 11.2, and 17.2 J/cm^2^ are enumerated in Table 3. For enamel tissues, it is clear that there are significant increases in the Ca/P in tandem with increasing the energy density. Whereas, no significant changes in the Ca/P ratios of dentin tissues were found in the energy densities of 5.6 and 11.2 J/cm^2^.

**Table 3:** Ca/P ratio for non-irradiated and irradiated enamel and dentin tissues at energy densities of 5.6, 11.2, and 17.2 J/cm^2^.

Dental tissue	Non-irradiated	5.6 (J/cm^2^)	11.2 (J/cm^2^)	17.2 (J/cm^2^)
Enamel	2.03 ± 0.11	2.19 ± 0.16^a^	2.97 ± 0.09^a^	3.16 ± 0.13^a^
Dentin	1.93 ± 0.06	1.93 ± 0.03	1.86 ± 0.08	1.82 ± 0.10^a^

Parameters (mean ± SD).

^a^ significant change to the non-irradiated values (p < 0.05).

## Discussion

From Fig 2(a) and 2(b), an increase in the peak area was observed with increasing incident energy density as the result was expected. Spectral line emissions of calcium started appearing clearly at an energy density of 3.5 and 2.5 J/cm^2^ for both enamel and dentin, respectively. The peak areas of calcium lines and energy densities were examined by data fitting for both enamel and dentin. The best fitting for enamel results was exponential fit, while the best fitting for dentin results was the linear one (Fig 2(a) and 2(b)). From best fitting, the interrupt points with X-axis were determined for all curves that represent ablation thresholds. These thresholds of Nd:YAG lasers in enamel and dentin were found to be 1.37, and 0.6 J/cm^2^, respectively. Enamel has higher thresholds than those of dentin. This may be attributed to the following reasons: enamel is composed of 85% mineral, 12% water, and 3% organic proteins. While, dentin is composed of 47% mineral, 33% protein mostly collagen, and 20% water. Dentin has a higher water content and less mineral density than enamel [18, 28]. ‏Therefore, ablated dentin must be avoided at a high energy density; because dentin contains organic materials and nerve endings that make it very sensitive [38]. Moreover, tooth dentin has abundant dentinal tubules, which make it as porous as sponge. In addition, the tubules provide the tissue elasticity and relatively low hardness, which would then result in the dentin’s lower ablation threshold with these lasers. Interestingly, the ablation threshold values that were found in this work were lower than those of other lasers reported in the published works [8, 10, 21–23, 28, 39]

From the SEM images, Enamel rod with the beginning of melting in some areas can be observed in Fig 4. In addition, the crater in the dentin was found to have a specific shape (Fig 4). By zooming more into the depth of the crater in the dentin, opened dentin tubules can be observed and no evidence of any melting trace. By comparing between Figs 3 and 4, even using a higher energy density with enamel than that was used for dentin with same number of shots (100 shots); enamel does not show a deep crater-like in dentin (Fig 4). ‏From these experiments, there were difficulties to obtain a homogeneous shape of the crater in enamel using low energies densities, so higher energy density was used. In fact, the enamel tissue is more calcified and it is much harder than dentin. Therefore, cracks might occur easily in enamel if high energy densities are used. Laser produced remarkable changes in the enamel rods. It altered their size and shape. Thus, enamel rods took‏ ‏variable shapes and irregular arrangements because the laser energy was converted into thermal energy when interacting with the enamel tissue. The melting and‏ ‏the following solidification process of the enamel produced a morphology that is characterized by columns and ‏ ‏separated by voids.

EDX quantitative analysis gives details about the elements atomic %. In dentistry, it is very difficult to conduct true quantitative analysis due to the fact that there is no standard reference for all types of samples like metallic, biological, etc. samples. For these cases, usually the peaks in sample spectrum are compared with the peak of the pure element. However, a semi-quantitative analysis can be conducted, if the SEM-EDX setup parameters such as applied voltage, magnification, spot size, etc.; are fixed. Long spectrum acquisition time was used in order to obtain a good signal to noise ratio with well-defined peaks and high number of counts. Even with these precautionary measures, some considerations must be taken like the relative error in the calculations, which is approximately 10% or more for the concentration of elements that are less than 5% [40].

From the data in Table 1, it is obvious that there were significant changes in Ca, P, O, and C atomic % of enamel tissue with all energy densities. Whereas, there were slightly changes in Ca, P, O, and C atomic % of dentin tissue (Table 2) with energy densities 17.2 J/cm^2^. The carbon atomic % is an important factor because the increase in carbon atomic % might indicate burning. This is due to the melting of sample surface, which increases when energy density increases. This could lead to burn the sample. The later finding is in full agreement with SEM results. However, perhaps the increases of carbon atomic % specifically in enamel are due to the formation of a carbonated apatite.

The results in Table 3, which were obtained from the EDX semi quantitative analysis, showed significant increase in Ca/P ratio of irradiated enamel tissue, especially at higher energy densities. It can be found when the energy density increased; the enamel showed a steady increase in the Ca/P ratio (Table 3). For dentin tissue, EDX semi quantitative analysis showed no significant changes in the Ca/P ratio of irradiated dentin tissues. The percentage of calcium and phosphorus after laser irradiation are very important. It was reported that the Ca/P ratio for human teeth varied from 1.92 (lowest in dentine) to 2.15 (highest in enamel) [41]. The calculated Ca/P ratio of non-irradiated enamel tissue is in the same range with those reported literatures [42–44]. Whereas, the calculated Ca/P ratio of non-irradiated dentin tissue is in the same range with those reported literatures [44–46]. Accordingly, the significant differences in the Ca/P ratios of enamel tissues indicate that the ablation using pulsed, 5 nanosecond, Nd:YAG laser at 1064 nanometer can change the chemical compositions of enamel tissue. On other hand, there were no significant differences in Ca/P ratio of dentin tissue using energy densities less than 5.6 J/cm^2^. Thus, these results indicate that these kinds of lasers can be used safely to ablate dentin tissue with lower energy densities.

## Conclusion

From this work, The ablation thresholds of enamel and dentin tissue using pulsed, 5 nanosecond, Nd:YAG laser at 1064 nanometer were found to be 1.37, and 0.6 J/cm^2^, respectively. In addition, the dentin ablation revealed lower ablation threshold than the ablation threshold of enamel. Appropriate laser energy densities for dentin ablation have been achieved without melting, carbonization, or micro-cracks. In addition, significant increase in the carbon atomic % and Ca/P ratios of enamel tissue were observed. Whereas, no significant changes in the carbon atomic % and Ca/P ratios of dentin tissue was detected using low energy densities. ‏Accordingly, these kinds of lasers are more useful for dentin ablation than for enamel ablation.
